# Supplementary material for: Urinary Eubacterium sp. CAG:581 Promotes Non-Muscle Invasive Bladder Cancer (NMIBC) Development through the ECM1/MMP9 Pathway
Source: Cancers (Basel). 2023 Jan 28;15(3):809. doi: 10.3390/cancers15030809 (PMC9913387; doi:10.3390/cancers15030809)
Supplement: Supplementary file 1 [file cancers-15-00809-s001.zip › cancers-2092900-supplementary.pdf]

**Urinary *Eubacterium sp. CAG:581* promotes non-muscle-invasive bladder cancer (NMIBC) development through ECM1/MMP9 pathway**

Yuhang Zhang, Wenyu Wang, Hang Zhou and Yimin Cui*

## Supplementary Tables

**Supplementary Table S1. Baseline information of NMIBC patients (n = 51) and healthy control participants (n = 47) in the Cohort 1.**

| Subgroup | Healthy Controls | NMIBC | HR (95%CI) |
|---|---|---|---|
| Total Number | 47 | 51 | 0.94 (0.84-1.01) |
| Age | | | |
| 30–39 | 21 (44.7%) | 19 (37.3%) | 0.71 (0.55-0.92) |
| 40–64 | 22 (46.8%) | 22 (43.1%) | 0.96 (0.78-1.05) |
| ≥65 | 4 (8.5%) | 10 (19.6%) | 1.29 (0.98-1.48) |
| Sex | | | |
| Female | 21 (44.7%) | 26 (51.0%) | 0.98 (0.84-1.04) |
| Male | 26 (55.3%) | 25 (49.0%) | 0.85 (0.69-1.01) |
| Smoking status | | | |
| Non | 30 (63.8%) | 29 (56.9%) | 0.82 (0.65-0.94) |
| Ex | 12 (25.6%) | 13 (25.5%) | 0.97 (0.74-1.09) |
| Current | 5 (10.6%) | 9 (17.6%) | 0.77 (0.54-1.09) |
| Alcohol consumption | | | |
| Never drinkers | 9 (19.1%) | 14 (27.5%) | 1.29 (0.83-1.36) |
| Up to 30 g/day | 20 (42.6%) | 22 (43.1%) | 1.09 (0.75-1.22) |
| More than 30 g/day | 18 (38.3%) | 15 (29.4%) | 0.67 (0.59-0.89) |
| Other clinical information | | | |
| BMI, kg/m2 | 21.94 | 23.45 | 0.88 (0.55-1.11) |
| Regularly exercise | 7 (14.9%) | 5 (9.8%) | 0.61 (0.44-0.84) |
| Hypertension | 20 (42.6%) | 25 (49.0%) | 0.99 (0.89-1.08) |
| Dyslipidemia | 29 (61.7%) | 20 (39.2%) | 0.78 (0.65-1.12) |

| | | | |
|---|---|---|---|
| Diabetes | 7 (14.9%) | 5 (9.8%) | 0.79 (0.60-1.04) |
| *Eubacterium sp. CAG:581* abundance | | | |
| *Eubacterium sp. CAG:581*-low/negative | 38 (80.9%) | 12 (23.5%) | 0.22 (0.15-0.32) |
| *Eubacterium sp. CAG:581*-high | 9 (19.1%) | 39 (76.5%) | 4.21 (2.54-5.33) |
| ECM1 Staining | | | |
| Positive | 28 (59.6%) | 12 (23.5%) | 0.37 (0.23-0.49) |
| Negative | 19 (40.4%) | 39 (76.6%) | 1.87 (1.61-2.83) |
| MMP9 Staining | | | |
| Positive | 36 (74.5%) | 16 (31.4%) | 0.42 (0.25-0.62) |
| Negative | 11 (25.5%) | 35 (68.6%) | 1.66 (1.49-1.88) |

**Supplementary Table S2. Baseline information of NMIBC patients (n = 406) and healthy control participants (n = 398) in the Cohort 2.**

| Subgroup | Healthy Controls | NMIBC | HR (95%CI) |
|---|---|---|---|
| Total Number | 398 | 406 | 0.99 (0.91-1.03) |
| Age | | | |
| 30–39 | 137 (34.42%) | 119 (29.31%) | 0.82 (0.81-1.01) |
| 40–64 | 201 (50.50%) | 209 (51.48%) | 0.99 (0.94-1.13) |
| ≥65 | 60 (15.08%) | 78 (19.21%) | 1.24 (0.97-1.23) |
| Sex | | | |
| Female | 181 (45.48%) | 186 (45.81%) | 0.98 (0.97-1.24) |
| Male | 217 (54.52%) | 220 (54.19%) | 0.96 (0.85-1.19) |
| Smoking status | | | |
| Non | 162 (40.70%) | 149 (36.70%) | 0.87 (0.79-1.01) |
| Ex | 121 (30.40%) | 109 (26.85%) | 0.85 (0.79-0.94) |
| Current | 115 (28.89%) | 148 (36.45%) | 1.23 (1.03-1.32) |
| Alcohol consumption | | | |
| Never drinkers | 186 (46.73%) | 199 (49.01%) | 1.02 (0.88-1.04) |
| Up to 30 g/day | 82 (20.60%) | 98 (24.14%) | 1.14 (1.11-1.43) |
| More than 30 g/day | 130 (32.66%) | 109 (26.85%) | 0.79 (0.73-1.04) |

| | | | |
|---|---|---|---|
| Other clinical information | | | |
| BMI, kg/m2 | 22.33 | 23.01 | 1.01 (0.85-1.09) |
| Regularly exercise | 87 (21.86%) | 65 (16.01%) | 0.70 (0.71-0.98) |
| Hypertension | 57 (14.32%) | 43 (10.59%) | 0.71 (0.73-1.01) |
| Dyslipidemia | 89 (22.36%) | 50 (12.32%) | 0.52 (0.51-0.83) |
| Diabetes | 28 (7.04%) | 45 (11.08%) | 1.55 (1.18-1.57) |
| *Eubacterium sp. CAG:581* abundance | | | |
| *Eubacterium sp. CAG:581*-low/negative | 309 (77.64%) | 60 (14.78%) | 0.16 (0.16-0.46) |
| *Eubacterium sp. CAG:581*-high | 89 (22.36%) | 346 (85.22%) | 3.78 (1.23-2.78) |

**Supplementary Table S3. Reagents and Materials**

| Reagent or Resource | Source | Identifier |
|---|---|---|
| **Antibodies** | | |
| Rabbit polyclonal anti-PTPN6 (SHP1) antibody | Abcam | Cat# ab227503 |
| Rabbit monoclonal anti-IKZF3 antibody | Abcam | Cat# ab139408 |
| Mouse monoclonal anti-β-actin antibody | Sigma-Aldrich | Cat# A5441 |
| Rabbit polyclonal anti-ECM1 antibody | Cell Signaling Technology | Cat# 83458S |
| Rabbit monoclonal anti-MMP9 antibody | Abcam | Cat# ab76003 |
| Rabbit monoclonal Anti-ERK1 (Phospho T202 + Y204) + ERK2 (Phospho T185 + Y187) antibody | Abcam | Cat# ab278538 |
| Rabbit polyclonal anti-ERK1/2 antibody | Abcam | Cat# ab196883 |

| | | |
|---|---|---|
| Rabbit polyclonal Anti-AKT1 + AKT2 + AKT3 (phospho S472 + S473 + S474) antibody | Abcam | Cat# ab192623 |
| Rabbit polyclonal anti-Anti-AKT1 + AKT2 + AKT3 antibody | Abcam | Cat# ab179463 |

**Chemicals and Reagents**

| | | |
|---|---|---|
| Ravoxertinib | Selleckchem | Cat# SCH772984 |
| Ulixertinib | Selleckchem | Cat# LY3214996 |
| poly-L-lysine/laminin | Merck | Cat# 25988-63-0 |
| DMEM | GIBCO | Cat# 11965-092 |
| Fetal bovine serum | GIBCO | Cat# 10099-141 |
| HBSS | Invitrogen | Cat# 14175103 |
| Rho-Kinase-IN-1 | MCE | Cat# 1035094-83-7 |
| BSA | Sigma-Aldrich | Cat# B2064 |
| β-Mercaptoethanol (BME) | Merck | Cat# 60-24-2 |
| Penicillin-streptomycin, liquid | Invitrogen | Cat# 15140122 |
| RIPA | BioVision | Cat# 2114-500 |
| LightCycler 480 SYBR Green | Roche | Cat# 4887352001 |
| FastQuant RT Super Mix Tiangen | Biotech | Cat# KR108 |
| ECL Western Blotting Substrate | Bio-rad | Cat# 1705060 |
| TRIzol | Invitrogen | 15596018 |

**Critical Commercial Assays**

| | | |
|---|---|---|
| Quant One Step qRT-PCR | Tiangen | Cat# FP303-01 |

| | | |
|---|---|---|
| Quant-iT PicoGreen dsDNA Assay Kit | Invitrogen | P11495 |
| Chromium Single Cell 30 Reagent Kits v3 | 10X Genomics | PN-1000092 |
| **Software and Algorithms** | | |
| SPSS 17.0 software | IBM | https://www.ibm.com/account/reg/ |
| GraphPad. Prism. v6.01 | GraphPad Prism | https://www.graphpad.com/ |
| 7500 Real Time PCR System | Applied Biosystems | N/A |
| ImageJ | NIH | https://imagej.nih.gov/ij/ |

**Supplementary Table S4. Primers for RT-qPCR assay**

| Genes | Forward (5'-3') primer | Reward (5'-3') primer |
|---|---|---|
| *Eubacterium sp. CAG:581* 16SrRNA | CCATCGCCATCGAGAGACAAG | CACGTAGTGCTTAGCATAGAGAG |
| *Ptpn6* | CTGGAGGTTCGACGTGAAG | TCCTGGCAGAAATAGGCTTTC |
| *Ikzf3* | CAGTTTCCATTCATCTTCCAAGG | CATCACCGTCGAGTCAGC |
| *Ecm1* | AGCCGTGTCATCGTCATG | CGGAGTTTGTATTCGGTTGTG |
| *Mmp9* | ACATTCTCCCCTTTGACCAC | CGATGTAGGTCTTAGCGTTCTC |
| *Gapdh* | ACTCCAAGGCCACTTATCACC | ATTGTTACCAACTGGGACGAA |

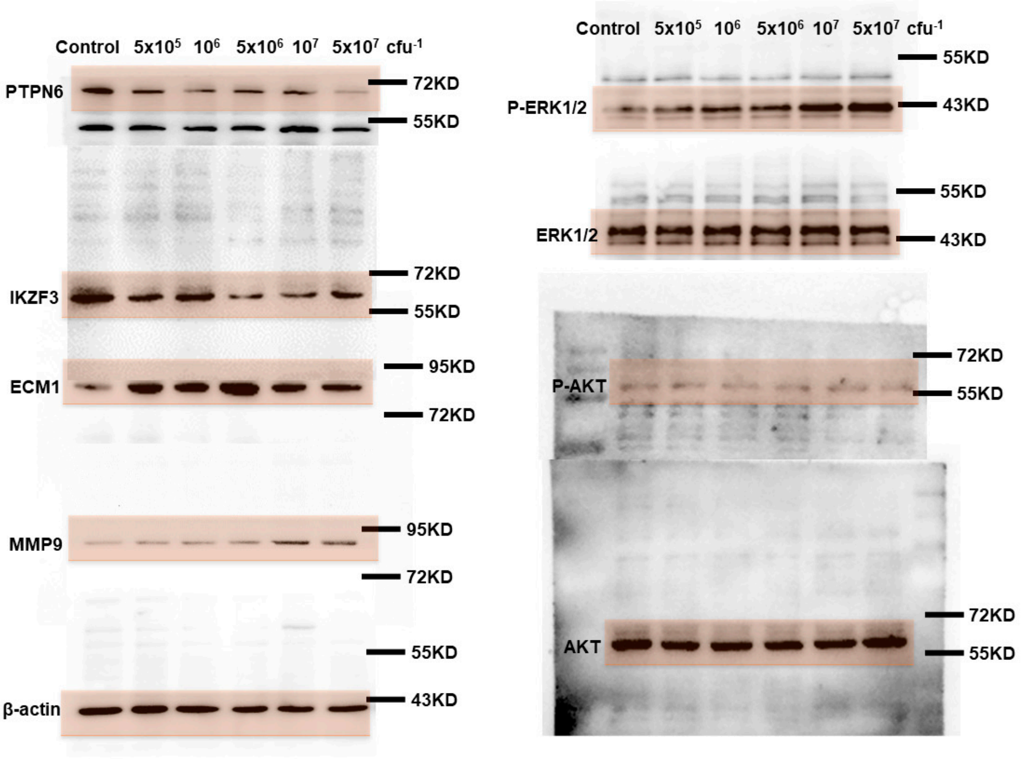


Figure 3B

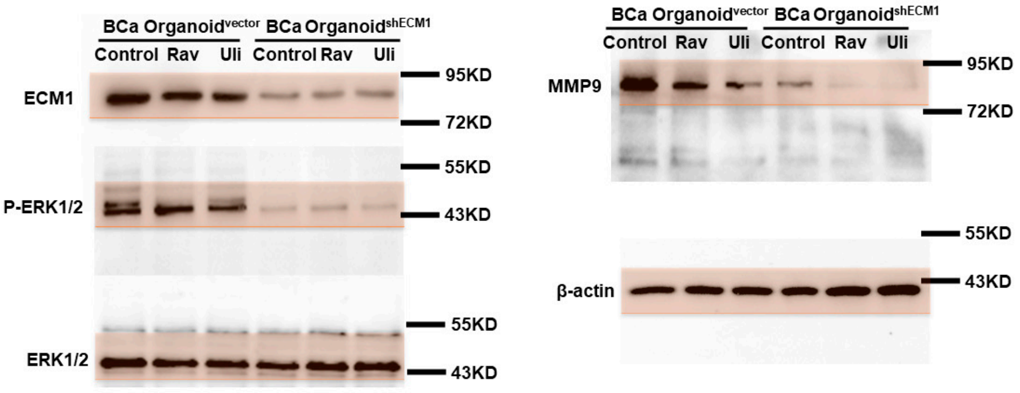


Figure 3C

**Supplementary Figure S1. Uncropped western blotting gels.**

A

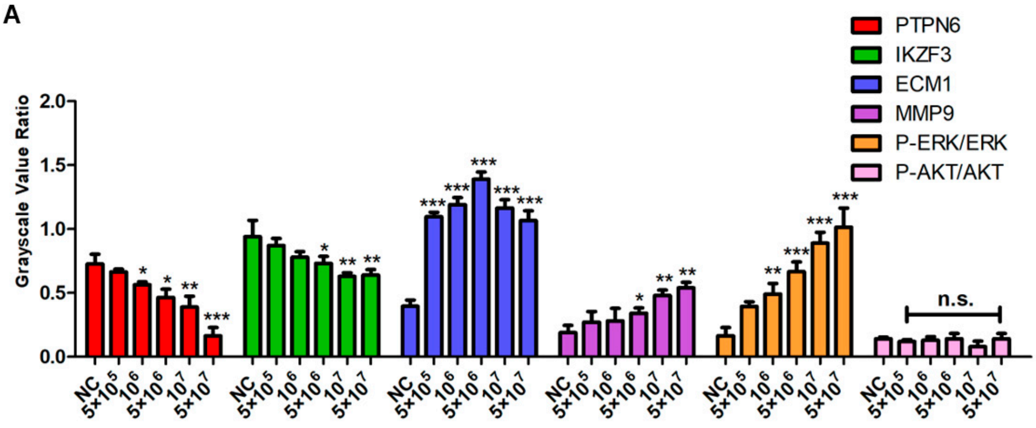


B

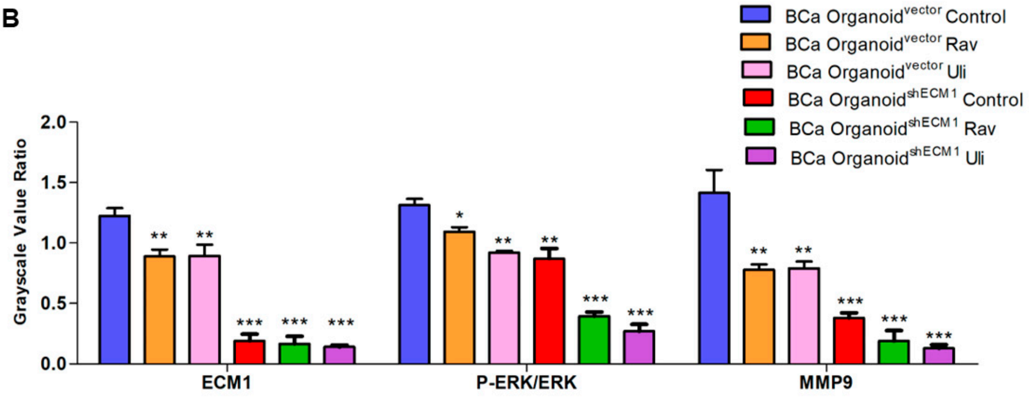


**Supplementary Figure S2. Quantified grayscale ratio for western blot bands of Figure 3B (A) and Figure 3C (B).**
